# Bladder calculi and ischemic bowel loop in association with inguinal sliding bladder hernia

**DOI:** 10.1093/jscr/rjag142

**Published:** 2026-03-17

**Authors:** Angel Arabadzhiev, Nikolay Y Yordanov, Manol Sokolov, Yavor Kyumyurdzhiyski, Tsvetan Popov

**Affiliations:** Clinic of Surgery, University Hospital “Alexandrovska”, 1 Georgi Sofiyski Street, Sofia, 1431, Bulgaria; Department of Surgery, Faculty of Medicine, Medical University – Sofia, 1 Georgi Sofiyski Street, Sofia, 1431, Bulgaria; Medical University – Sofia, bul. “Acad. Ivan Geshov” № 15, Sofia, 1431, Bulgaria; Clinic of Surgery, University Hospital “Alexandrovska”, 1 Georgi Sofiyski Street, Sofia, 1431, Bulgaria; Department of Surgery, Faculty of Medicine, Medical University – Sofia, 1 Georgi Sofiyski Street, Sofia, 1431, Bulgaria; Department of Radiology, Medical University – Sofia, 1 Georgi Sofiyski Street, Sofia, 1431, Bulgaria; Clinic of Surgery, University Hospital “Alexandrovska”, 1 Georgi Sofiyski Street, Sofia, 1431, Bulgaria; Department of Surgery, Faculty of Medicine, Medical University – Sofia, 1 Georgi Sofiyski Street, Sofia, 1431, Bulgaria

**Keywords:** sliding hernia, bladder calculi, inguinoscrotal hernia, TAPP repair, open hernia repair, strangulated hernia

## Abstract

Inguinal bladder hernia is a rare entity, accounting for 1%–4% of inguinal hernias, and the presence of bladder calculi within the herniated bladder is exceptionally uncommon. Preoperative imaging is essential to ensure diagnosis and prevent intraoperative complications. A 61-year-old male presented with abdominal pain, urinary symptoms, and a progressively enlarging left inguinoscrotal mass. Computed tomography demonstrated an incarcerated inguinoscrotal hernia containing a portion of the urinary bladder and small bowel. Laparoscopic transabdominal preperitoneal repair was initiated but converted to an open procedure due to bowel ischemia. A subtotal sliding bladder hernia with a non-iatrogenic bladder wall defect containing two calculi was identified. Cystorrhaphy and small bowel resection with stapled anastomosis were performed, followed by Bassini hernia repair. The postoperative course was uneventful. Inguinal bladder hernia with bladder calculi is extremely rare. Preoperative imaging is crucial for surgical planning, and an open approach may be preferable when bladder stones or strangulated bowel are present.

## Introduction

Inguinal bladder hernia (IBH) is a rare entity, comprising 1%–4% of all inguinal hernias . The condition was first documented in the 16th century by Felix Platter and Dominic Sala [1]. It may involve a small portion of the bladder, a diverticulum, or the entire organ [2]. Massive herniation of the bladder into the scrotum was termed “scrotal cystocele” by Levine in 1951, who identified only 30 prior cases [3]. To date, more than 100 cases of scrotal cystocele have been reported. This report aims to characterize the condition, outline potential complications, and examine how preoperative suspicion, imaging diagnosis, and patient-specific factors influence surgical management and approach.

## Case report

A 61-year-old male presented with nausea, vague periumbilical abdominal pain, decreased urinary output, sensation of incomplete bladder emptying, and suprapubic discomfort relieved by urination—symptoms present for 1 week. He reported a longstanding minor left inguinal bulge that had progressively enlarged over the preceding week to extend into the scrotum. Medical history was notable for a previously repaired right inguinal hernia.

On examination, a left irreducible inguinoscrotal hernia was found, without signs of peritonitis (no guarding, rigidity, or rebound tenderness). Bowel sounds were normal, and there was no costovertebral angle tenderness. Abdominal X-ray showed no free air or air-fluid levels.

**Figure 1:**
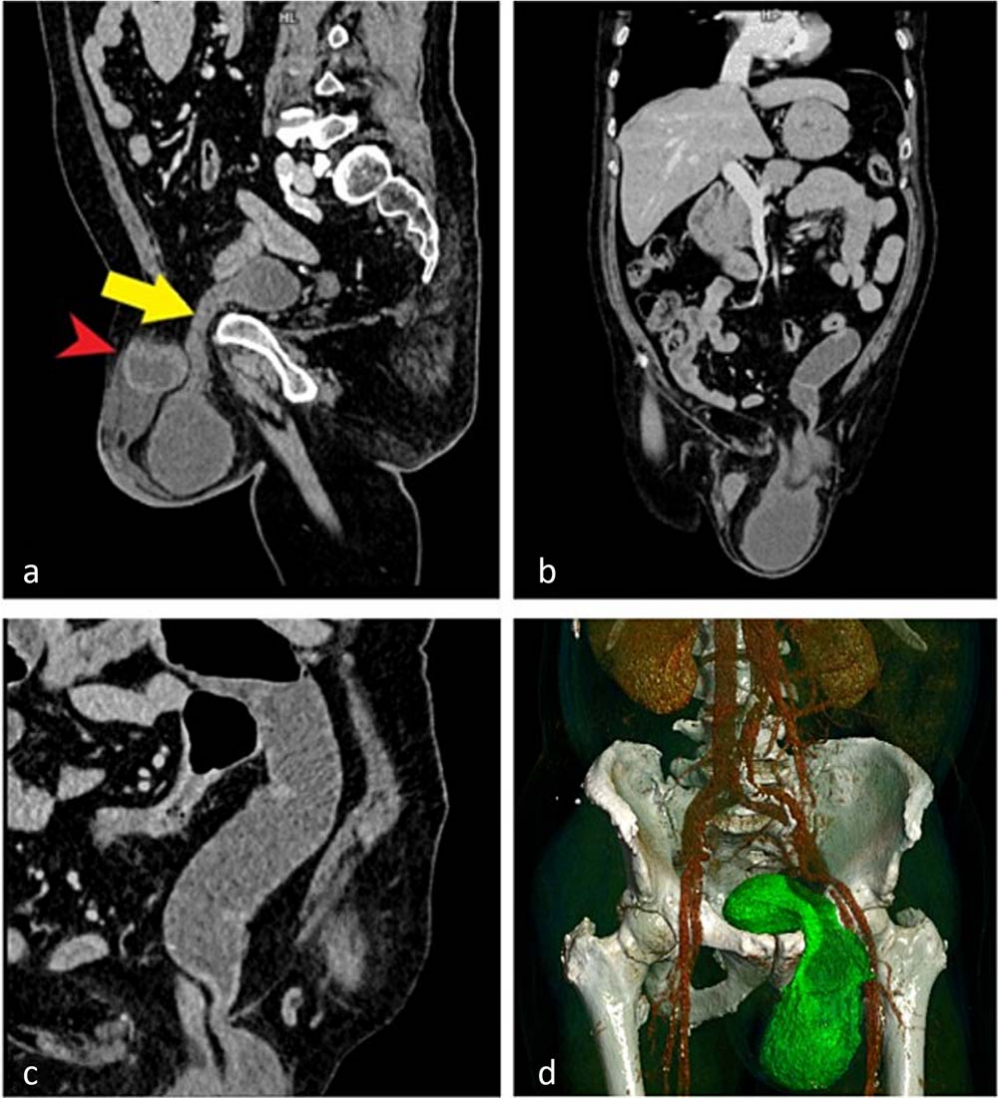
CT images demonstrating an incarcerated left inguinoscrotal hernia. (a) Sagittal plane showing herniation of the anterior bladder wall and a small bowel loop. (b) Coronal plane view of the herniated portion of the urinary bladder and small bowel. (c) Dilated small bowel loop with an air–fluid level proximal to the site of obstruction. (d) Three-dimensional reconstruction illustrating the herniated bladder.

Computed tomography (CT) demonstrated an incarcerated left inguinoscrotal hernia containing a portion of the urinary bladder, a small bowel loop, and free fluid within the hernial sac (Fig. 1).

**Figure 2:**
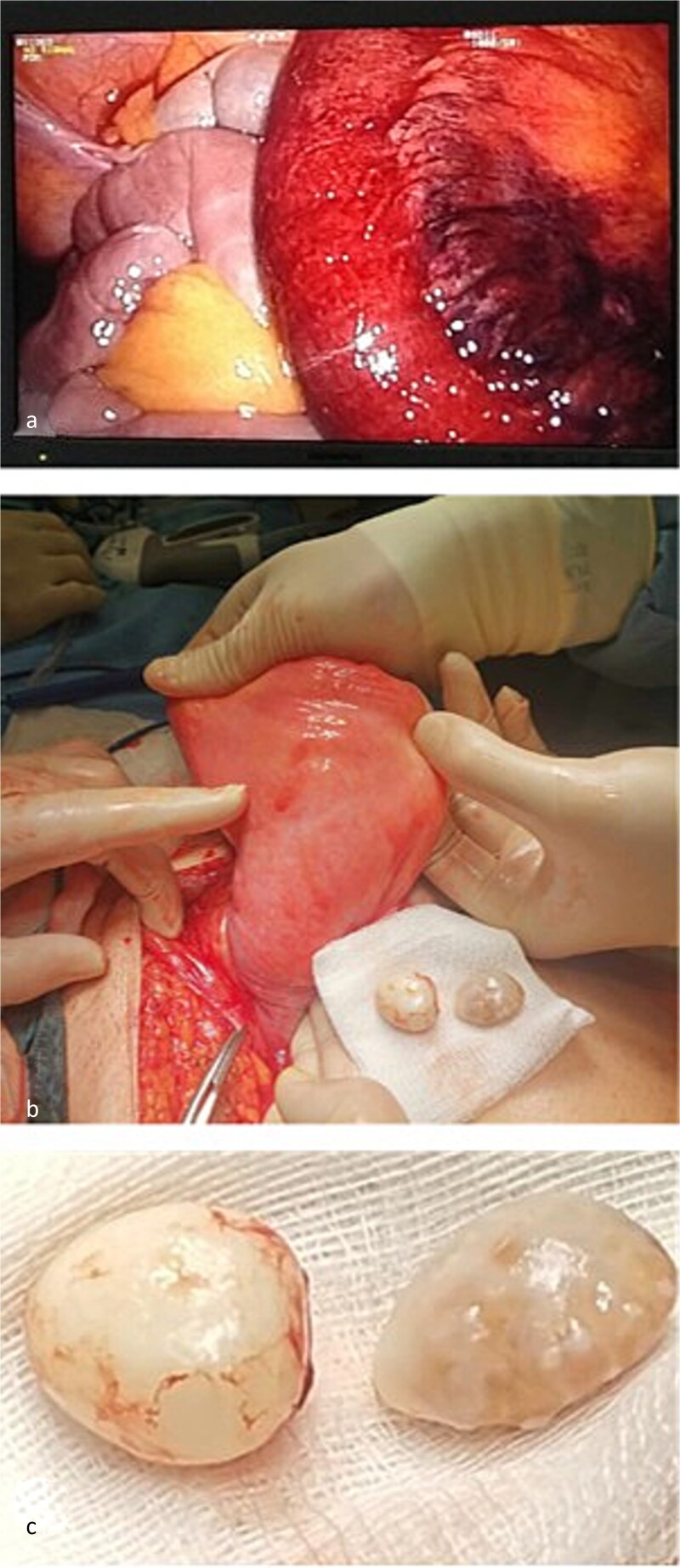
Intraoperative findings. (a) Ischemic small bowel loop identified during initial laparoscopic inspection. (b) Urinary bladder after reduction of the hernia and separation from the cord structures. (c) Two bladder calculi removed through a non-iatrogenic bladder wall defect.

A laparoscopic transabdominal preperitoneal (TAPP) repair was initiated using Veress needle insufflation. An 11-mm optical trocar was placed infra-umbilically, with two 5-mm working trocars along the midclavicular lines. Initial inspection revealed an ischemic small bowel loop (Fig. 2a), leading to immediate conversion to open surgery.

The peritoneal cavity was desufflated, and a left para-inguinal incision was performed. The hernial sac contained the ischemic bowel and a sliding subtotal prolapse of the bladder, positioned postero-medially. A non-iatrogenic bladder wall defect was identified, through which two calculi were extracted (Fig. 2c). Cystorrhaphy was performed with 2-0 Vicryl sutures. Careful dissection separated the sac and sliding bladder from the spermatic cord (Fig. 2b). The hernia was reduced, the ischemic bowel segment resected, and continuity restored with side-to-side stapled anastomosis. The posterior wall of the inguinal canal was reinforced using the Bassini technique with non-absorbable Prolene 1 sutures. No prosthetic mesh was placed due to the clean-contaminated field.

The postoperative course was uncomplicated, and the patient was discharged on day 8. Histopathology of the resected bowel confirmed vascular congestion, mucosal erosions, necrosis, submucosal edema, and neutrophilic infiltration. The bladder calculi showed peripheral fibrosis with a core of fat, cholesterol crystals, and dystrophic calcifications.

## Discussion

Development of scrotal cystocele is multifactorial, involving bladder outlet obstruction (e.g. benign prostatic hyperplasia, tumors, cystolithiasis, bladder neck dysfunction), elevated BMI, reduced bladder compliance, and weakened pelvic floor musculature—explaining predominance in elderly males [4].

Preoperative CT is advised for males over 50 with lower urinary tract symptoms (LUTS) or prior hernia repair to minimize risk of inadvertent bladder injury [5]. Older literature reported preoperative diagnosis in only 7% of cases, with most discovered intraoperatively; contemporary series show ~77% preoperative identification, attributable to routine advanced imaging [6, 7].

Small IBHs may be asymptomatic, while massive inguinoscrotal hernias often present with nonspecific LUTS secondary to partial obstruction. Three anatomical variants exist: paraperitoneal (most common; bladder medial to peritoneum), intraperitoneal (bladder fully covered by peritoneum), and extraperitoneal (bare bladder herniation) [8].

Historically, open repair predominated. In our reviewed cases, laparoscopic (58%) and open (42%) approaches were comparable; among minimally invasive repairs, totally extraperitoneal (TEP) was used in 82% and TAPP in 18%. Tantia advocates TAPP for sliding hernias, including IBH [9]. One septic case employed staged TAPP reduction followed by open mesh hernioplasty to avoid contamination [10].

Bladder calculi coexisted in 37% of reviewed cases. Only 11 prior reports of vesicolithiasis in IBH exist; this represents the 12th. Stones likely arise from urinary stasis in the herniated portion, though primary lithiasis remains possible [11].

All previously reported vesicolithiasis cases underwent open repair, with calculi visible preoperatively. However, stones may not always be radiographically apparent, necessitating clinical vigilance. No conversions were noted in our reviewed laparoscopic series, contrasting Tantia’s 10.8% conversion rate for irreducible complete sliding hernias [9]. Bladder calculi can cause irreducibility, favoring open access [12]. Recent meta-analysis supports open repair when bowel obstruction or strangulation is present [13].

Hernia size was documented in only eight cases, with no meaningful difference between approaches (one outlier excluded). Operative time was reported sparsely (mean 110 min laparoscopic vs. 80 min for one open bilateral repair) [14], limiting comparative conclusions.

## Conclusions

Sliding inguinal bladder hernia with associated vesicolithiasis is exceptionally rare. Preoperative imaging is essential in patients with LUTS or risk factors to avoid intraoperative complications and optimize surgical strategy. Laparoscopic repair is feasible and safe in selected cases. However, confirmed bladder calculi or incarcerated/strangulated bowel should prompt consideration of an open approach.
